# Polyelectrolyte Nanoparticles of Amphiphilic Chitosan/Pectin from Banana Peel as Potential Carrier System of Hydrophobic Molecules

**DOI:** 10.3390/polym12092109

**Published:** 2020-09-16

**Authors:** Paula A. Méndez, Betty L. López

**Affiliations:** 1 Grupo de Investigación en Etnofarmacología, Productos Naturales y Alimentos, Escuela de Ciencias Básicas Tecnología e Ingeniería, Universidad Nacional Abierta y a Distancia, Calle 14 Sur # 14-23, Bogotá 111511, Colombia; 2 Grupo de Investigación Ciencia de los Materiales, Instituto de Química, Facultad de Ciencias Exactas y Naturales, Universidad de Antioquia, Calle 70 N° 52-21, Medellín 050010, Colombia; bettylope@gmail.com

**Keywords:** carbohydrate, chitosan, encapsulation, extraction, nanoparticles, pectin, polyelectrolyte

## Abstract

In this study, pectins were extracted from banana wastes *Musa paradisiaca* under different acidic conditions, obtaining pectins with different degrees of esterification (DE) depending on the acid type and pH. The formation of the polyelectrolyte nanoparticles was evaluated according to the DE of the pectin, the mass ratio of the polymers of pectin to amphiphilic chitosan (AmCh), and their concentration. The properties of the polyelectrolyte nanoparticles were evaluated at different pH and temperatures. The pectin with 24.3% DE formed polyelectrolyte nanoparticles through the electrostatic interaction with AmCh, which was evidenced by changes in the zeta potential and particle size. The study of mass ratio AmCh:Pectin, to get a stable system, showed that it must be at least equal (1:1), or AmCh must be in higher proportion (6:1, 50:1, 100:1), and the polymers concentration must be 1 mg/mL. The study of the temperature effect showed that, when the temperature increases, the particle size decreases, and the pH study showed a stable particle size for the polyelectrolyte nanoparticles in the range of pH 5–6. Nile Red (NR), a hydrophobic molecule, was encapsulated in the polyelectrolyte nanoparticles with a loading capacity of 1.8% and an encapsulation efficiency of 80%.

## 1. Introduction

Food waste has attracted attention in recent years due to the negative impact generated towards the environment. In 2016, the Food and Agriculture Organization (FAO) reported a production of food wastage of 1.6 billion tons per year at the global level. For that reason, it has been important to study the re-use of wastes [1]. According to FAO reports in 2013, Colombia is the third largest exporter of organic bananas in the world, which implies that the consumption of this product is very high, generating large amounts of peel waste [2]. Banana peels *Musa paradisiaca* has been studied for pectin extraction using acid hydrolysis [3,4,5,6,7]. The properties of the pectins depend on the extraction method, source, and preparation of the material vegetable [8,9]. Pectin is a heteropolysaccharide which is mainly composed of linear chains of partially methyl-esterified (1,4) α-d-galacturonic acid (GA) residues, and three domains are mainly identified in the pectic macromolecule: homogalacturonan (HG), rhamnogalacturonan I (RGI), rhamnogalacturonan II (RGII) [10]. According to the degree of esterification (DE) of the carboxyl groups of the d-galacturonic acid, pectins are classified as high-esterified pectins (DE > 50%) and low-esterified pectins (DE < 50%) [1,11].

Most studies have focused on the formation of the polyelectrolyte complexes based on chitosan and pectin extracted from citrus fruit; specifically the characterization of the polyelectrolyte nanoparticles when evaluating the effect of the polymer concentration, order of addition, the mass mixing ratio, storage, and pH of the solution; to address the use in the treatment of chronic wounds and other applications [12,13,14]. On the other hand, chitosan–pectin complexes have shown resistance to UV action higher than homopolymers, which is a desirable property in medical or pharmaceutical applications [15]. A few research papers have been focused on the encapsulation process using the pectin polymer, as for example the polyelectrolytes of pectin–chitosan to encapsulate insulin [16], zein–pectin nanoparticles to carry up curcumin [17], and pectin–chitosan-tripolyphosphate nanoparticles to encapsulate the herbicide Paraquat [18]. Our study complements previous works by an evaluation of DE of the pectin in the polyelectrolyte formation, the temperature effect on the stability of the polyelectrolytes based on amphiphilic chitosan (AmCh) and pectin, and the capacity of the polyelectrolytes to encapsulate the Nile Red (NR), a solvatochromic fluorophore with different behavior in polar and no polar environment. Results indicate that the polyelectrolyte systems of AmCh:Pec have potential as a reservoir of hydrophobic molecules.

Pectin was extracted from banana peel *Musa paradisiaca* using two types of acid (citric acid and hydrochloric acid) and pH (1 and 2) to evaluate the properties of the pectin obtained, such as yield, degree of esterification and thermal decomposition. From these, the higher yield was obtained with citric acid, and pectin with low DE was obtained with hydrochloric acid. Pectin with a low DE of 24.3% produced smaller particle sizes with average value of 370 nm. The mass ratio and concentration of the polymers and the effect of the temperature and pH were evaluated to find that the mass ratio of the polymers must be equal (1:1), or the AmCh polycation must be in higher proportion, and the optimal polymers concentration is 1 mg/mL. The particle size decreased when the temperature increases up to 40 °C and the systems were more stable at pH 5–6. Finally, the polyelectrolyte nanoparticles AmCh:Pectin showed capacity to encapsulate a hydrophobic molecule. Nile Red (NR) was used as the proof molecule, obtaining a loading capacity of 1.8% and encapsulation efficiency of 80%.

## 2. Materials and Methods 

### Materials

Banana peel *Musa paradisiaca* was provided by local fruit market in Bogotá, Colombia. Chitosan (Ch) with low molecular weight (*M*_w_ = 5 kDa) and degree of deacetylation (DD) of 76 % was used. This polymer was obtained by oxidative degradation with sodium nitrite from chitosan with *M*_w_ = 150 kDa (Supplied by PolyScience Inc., 400 Valley Road Warrington, PA, USA), and it was characterized according with our previous work [19]. This molecular weight was selected due to the chemical modification with OA is favored by the carbodiimide chemistry route, and it work can complement another study where hyaluronic acid was used as polyanion to interact with AmCh nanoparticles [20]. Sodium nitrite, Nile Red (NR) and 1-ethyl-3-(3-dimethylaminopropyl) carbodiimide (EDC) were purchased from Sigma Aldrich, 3050 Spruce Street, St. Louis, MO 63103 USA. Acetic acid, hydrochloric acid 37%, citric acid, acetone, sodium hydroxide, acetonitrile, ethanol, and sodium acetate were supplied by Merck KGaA, 64271 Darmstadt, Germany. Commercial oleic acid (OA) and sodium hypochlorite (NaOCl, 0.01%) were used.

1. Pectin extraction from banana peel *Musa paradisiaca*

The banana peels *Musa paradisiaca* were washed with sodium hypochlorite 0.01% and water to eliminate any impurities or microorganisms. These were then cut into small squares and 300 g of peel were pretreated in 1 L of distilled water at 90 °C for 10 min in order to deactivate the pectic enzymes which are responsible of the hydrolysis of methyl ester groups in pectin [21]. After that, the banana peels were filtered with canvas cloth and dried at 50 °C until constant weight of raw material (134 g). The dried banana peels were pulverized and stored in a desiccator for an approximate period of eight months.

2. Effect of acid type and pH on the pectin properties

The pectin extraction was carried out by acid hydrolysis of the raw material. The experimental design considered two variables: pH at two levels (1.0, 2.0) and two acid types: inorganic hydrochloric acid, HCl and organic citric acid, CA. The conditions were selected in accordance with studies which have shown higher extraction yields with organic acids such as CA and higher DE with HCl [4,22]. Low pH ranges allow greater cell wall breakdown [22]. The samples were named Pec_HClpH1_, Pec_HClpH2_, Pec_CApH1_, and Pec_CApH2_. Briefly, 50 g of the pulverized material were treated with 1L of acid solution (HCl), and (CA), both with concentration 0.1 M. The mixture was heated at 85 °C for 1 h [23,24]. After this time, it was filtered with canvas cloth. The filtrate was cooled in an ice bath for 15 min and then it was centrifuged for 10 min at 3000 rpm. The pectin was precipitated from supernatant with 95% ethanol, in a volume ratio 1:2 (supernatant: ethanol). The precipitate remained for 4 h in an ice bath and then the pectin was filtered with canvas cloth. Pectin gel was washed with ethanol 70% until pH 6–7. Finally, the pectin gel was dried at 50 °C until constant weight and stored in a desiccator. Pectin was characterized by FTIR (Fourier transform infrared), thermogravimetric analysis (TGA), the percentage of the degree of esterification (%DE) was calculated by band deconvolution from FTIR and the extraction yield was calculated according to Equation (1) [25], where *W* is the weight of the extracted pectin after drying and *W*_0_ is the weight of the pulverized material (50 g).

(1) $$Extraction\;yield\;\left(\%\right)=\frac{W}{W_{0}}*100$$

The pectin FTIR spectra were obtained in the range 4000–450 cm^−1^. The samples were dispersed in KBr and the transmittance recorded over 12 scans in a FTIR Perkin Elmer spectrum one (instrument distributed by PerkinElmer, USA). The degree of esterification (DE) was determined by the area’s relation of the absorption bands at 1745 cm^−1^ (methyl esterified carboxylic group) and 1633 cm^−1^ (carboxylic ion) according to previous method [1]. This IR analysis was done using the same concentration and thickness in each sample.

Thermogravimetric analysis (TGA) was performed in a thermogravimetric analyzer Q-500 TA instruments (instrument distributed by Lanzetta Rengifo & CIA Ltd., Bogotá, Colombia), using approximately 7 mg of polymer pectin to stabilize the thermal properties. The thermograms were recorded from 30 to 750 °C at a scanning rate of 10 °C/min under a nitrogen gas atmosphere.

3. Preparation and characterization of polyelectrolyte nanoparticles based on AmCh:Pec

AmCh polymer was prepared from low molecular weight chitosan modified with oleic acid (12%) through the formation of amide linkages by the carbodiimide chemistry route [19]. The polyelectrolyte nanoparticles were prepared using AmCh polymer and the pectin isolated from banana (*Musa paradisiaca*) peels through sonication as a homogenization method using a probe-type sonicator (SONICS, VCX130 vibra-cell) at 130 W and 20 kHz in an ice bath where an acetate buffer 0.2 M with pH 4.6 and 7% (*v*/*v*) ethanol, due to the solubility of the AmCh polymer, was used. The sonication used a pulse function (amplitude 90%; pulse on 20 s; pulse off 20 s) during 2 mins. 

The particle size was characterized by dynamic light scattering (DLS) (HORIBA LB-550, 1-7-8 Higashi-Kanda Chiyoda-ku, Tokyo, Japan) in a HORIBA LB-550 (instrument distributed by S&S Ingeniería, Bogotá, Colombia) and the surface charge was obtained by zeta potential in a Malvern, Zetasizer Nano Z (instrument distributed by Cecoltec, Medellín, Colombia). The polydispersity index (PdI) was measured by dynamic light scattering (DLS). The measurements were determined in triplicate using freshly prepared samples dispersed in MilliQ-water.

In order to establish the conditions to form polyelectrolyte complex nanoparticles AmCh:Pec, the effect of mass ratio and concentrations of AmCh and Pectin, pH and temperature were evaluated.

4. Effect of mass ratio of AmCh:Pec

AmCh polymer and pectin were added to acetate buffer 0.2 M with pH 4.6 with 7% (*v*/*v*) of ethanol, in different mass ratio (AmCh:Pec 1:1, 1:2, 1:3, 1:6, 2:1, 3:1, 6:1, 50:1, 100:1). The polymer solutions were mixed and sonicated under the same conditions described previously.

5. Effect of the AmCh and pectin concentration

According with the results of the effect of the mass ratio, the system with smaller particle size and homogeneous size distribution was selected. To evaluate the effect of two polymer concentrations, the experimental design was considered: AmCh and pectin concentrations at three levels, 1.0, 1.5, and 2.0 mg/mL, and 1.0, 0.5, and 0.2 mg/mL, respectively. The polymer solutions were mixed and sonicated under the same conditions described previously.

6. Effect of the pH and temperature

To evaluate the effect of pH and temperature on the formation of the systems, the polyelectrolyte AmCh:Pec nanoparticles were prepared under the same conditions described previously. The changes in the particle size and polydispersity index of the AmCh:Pec nanoparticles were measured by DLS for the systems that were kept at different temperatures: 25, 30, 35, 37, and 40 °C and at different pH values (2.0–7.5).

7. Morphology of the polyelectrolyte nanoparticles

The morphology of the optimized nanoparticles was determined by scanning electron microscopy (SEM, DENTON VACUUM Desk IV). The sample was prepared by adding a drop on the carbon tape, and then it was dried under vacuum for 24 h. The sample was covered with gold and then analyzed with the software ImageJ.

8. Encapsulation of Nile Red (NR) in polyelectrolyte nanoparticles based on AmCh:Pec

To study the encapsulation behavior, a proof molecule was used. Nile red was encapsulated in polyelectrolyte nanoparticles using three different mass ratios with respect to the total weight of the polymers (2.0%, 1.0%, 0.5%). Briefly, AmCh was added in an acetate buffer 0.2 M at pH 4.6, with a final concentration of 1 mg/mL; and pectin with final concentration of 1 mg/ml. NR was dissolved in 1 mL of ethanol and it was added in the polymer’s mixture. The nanoparticles were prepared using the same conditions described previously by sonication. The nanoparticles were purified by ultrafiltration using a regenerated cellulose membrane of 1 kDa and a Stirred Ultrafiltration Cell, Merck Millipore Ref. 5122 equipment. Nanoparticles solution was washed with ethanol 7% (*v*/*v*) to eliminate unencapsulated NR. Finally, nanoparticle solutions were recovered in MilliQ water.

To quantify the NR encapsulated, the washes were analyzed through the calibration curve and compared with the amount of NR encapsulated in the nanoparticles. For the last one process, the dispersions of the nanoparticles were lyophilized for one day. The solid obtained was dissolved in a known volume of acetonitrile and an aliquot of this solution was taken to be analyzed by UV-vis at 537 nm and compared with the NR reference curve [26]. NR encapsulation efficiency was determined in triplicate and the values were reported as mean ± standard deviation (SD). The encapsulation efficiency (EE) and loading capacity (LC) were calculated using the Equations (2) and (3), respectively. In addition, AmCh:Pec nanoparticle sizes were analyzed by DLS technique after the encapsulation process.

(2) $$\%EE\;\left(Encapsulation\;efficiency\right)=\frac{\left[Amount\;of\;NR\;within\;nanoparticles\right]}{\left[Total\;NR\;added\right]}\times 100$$

(3) $$\%LC\;\left(Loading\;capacity\right)=\frac{\left[Amount\;of\;NR\;within\;nanoparticles\right]}{\left[Total\;amount\;of\;nanoparticles\right]}\times 100$$

9. Statistics

All data are presented as mean ± standard deviation (± SD) from at least three measurements. Means are compared between groups by one-way analysis of variance (ANOVA) and two groups by Student’s t test. A *p* < 0.05 are considered statistically significant. Calculations were done using the software Statgraphics Centurion XVI.I

## 3. Results and Discussions

### 3.1. Pectin Extraction and Characterization

**Figure 1:**
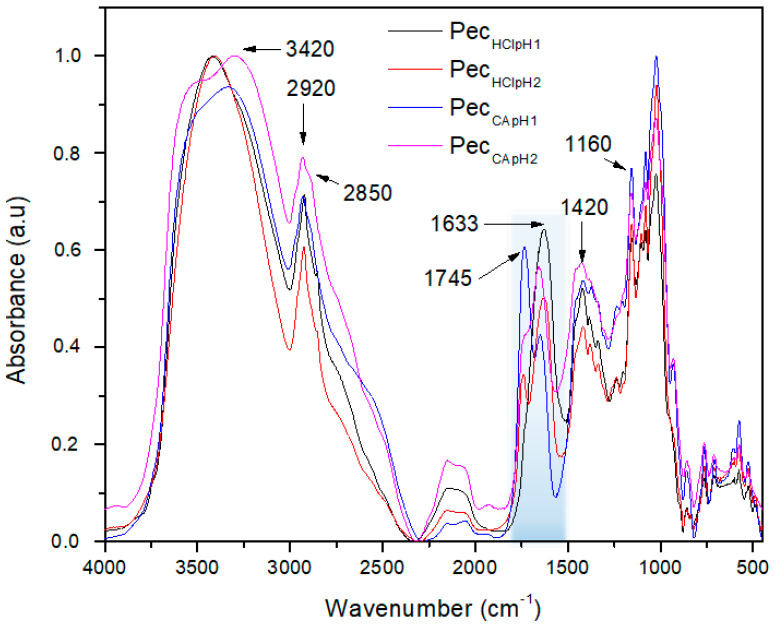
FT-IR spectra of pectin samples: **----** Pec_HClpH1_, **----** Pec_HClpH2_, **----** Pec_CApH1_, **----** Pec_CApH2_.

Figure 1 shows the infrared spectra of the extracted pectins from banana peels *Musa paradisiaca* at the different pH and acid medium. In general, it was observed at 3420 cm^−1^ the characteristics absorption peaks of –OH stretching hydroxyl group, which can be overlapped with hydroxyl group of carboxylic acid present in network of the pectin. At 2920 and 2850 cm^−1^ appears the stretching band of the aliphatic group –CH_2_. The band at 1745 cm^−1^ was assigned to stretching of carbonyl group of the ester, –COOCH_3_ and the band at 1633 cm^−1^ is the stretching of carboxylate group, –COO^−^. The signal in 1420 cm^−1^ was assigned to the flexion of the C–H bonding. The region of 1149–1020 cm^−1^ was referred to the characteristic bands of stretching of C–O–C bonding [15,27,28,29]. 

**Figure 2:**
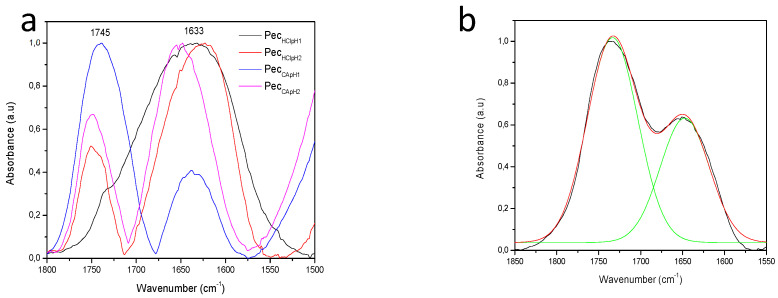
(**a**) Base line subtracted samples in the region 1800-1500 cm^−1^ and (**b**) an example of the band deconvolution of the signals in 1747 cm^−1^ (–COOCH_3_) and 1630 cm^−1^ (–COO^−^) for the Pec_CApH1_ sample.

Figure 2a shows the region between 1800–1500 cm^−1^ shaded in Figure 1. This demonstrates in a qualitative way that the sample Pec_CApH1_ (line **----**) presented a higher degree of esterification because the band at 1745 cm^−1^ had a higher intensity in comparison to the other samples. In addition, Pec_CApH2_ (line **----**) and Pec_HClpH2_ (line **----**) presented a similar DE, and Pec_HClpH1_ (line **----**) did not show significant number of esterified groups. According to previous studies, the DE can be calculated by the area relation of the bands at 1745 cm^−1^ and at 1633 cm^−1^ [15,27,28,29]. For that reason, the base line was subtracted for all samples as shown in the Figure 2a, then each area under the curve was obtained by band deconvolution with the Origin program, shown in Figure 2b for the Pec_CApH1_ sample. The results of the area relation are described in a later section.

**Figure 3:**
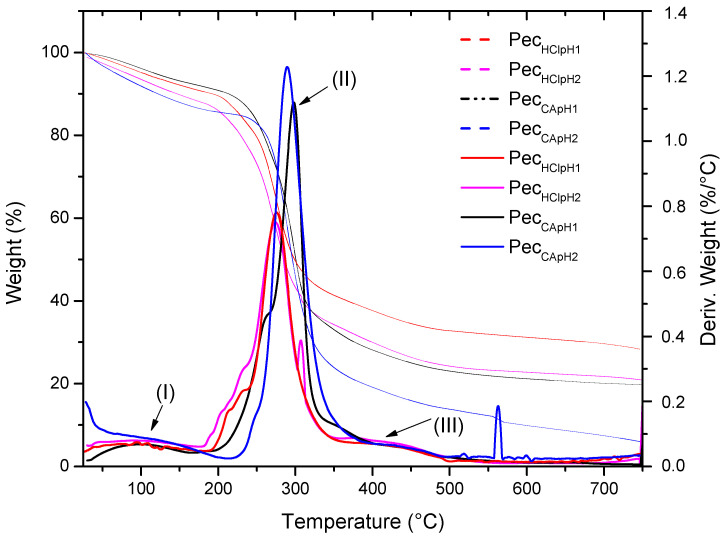
Typical thermogravimetric curves (weight loss vs. temperature) and (first derivative vs. temperature) for pectin extracted with inorganic and organic acid: **----** Pec_HClpH1_, **----** Pec_HClpH2_, **----** Pec_CApH1_, **----** Pec_CApH2_.

Figure 3 shows the thermogravimetric curves of pectin extracted with inorganic and organic acid. The first weight loss (I) was attributed to physiosorbed water, which was in the range 85–100 °C. The second loss (II) was associated to the pyrolysis process or thermal decomposition of the pectin [15,30]. The pectin extracted with inorganic acid showed a decomposition temperature close to 274 °C and pectin extracted with organic acid at 298 °C. The third weight loss (III) was attributed to byproduct formation after pyrolysis of the pectin [31].

### 3.2. The Effect of Acid Type and Ph on Extraction Yield and Degree of Esterification of Pectin

Most of the studies have shown higher extraction yields with organic acids as the citric acid (CA), from different extraction sources such as cubiu (*Solanum sessiliflorum*) fruits, [32] orange lime residues, [33] grape pomace, [27] cocoa husks, [22] and others. Our results showed coherence with these authors, the Table 1 shows the yields obtained using hydrochloric acid and citric acid, these results demonstrated that the extraction yield depends only on the acid type and the pH variable did not have a significant effect. The pectin extracted from banana peel *Musa paradisiaca* presented a higher yield with citric acid, nearly (20%), than the one extracted with hydrochloride acid (7%). This behavior is due to the drastic conditions of the hydrolysis of inorganic acids. The mineral acids can easily break the vegetable membrane allowing the pectin extraction. However, at the same time the pectin is submitted an extensive acid hydrolysis forming smaller polymer chains which do not precipitate with ethanol [32]. From an environmental point of view and by extraction yield, the use of organic acids is a better alternative to get pectin polymer from different vegetable wastes. In addition, the DE, that is one of the most important properties of pectin, is affected by the acid type. In the present study, the DE was determined by potentiometric titration, however, the results did not show coherence. Pectin presented gelling properties [34] which carried out to the formation of colloids during the titration process, so, the methoxy groups could be oriented inside of the colloid and the carboxyl groups oriented outside, simulating an amphiphilic behavior which did not permit the adequate quantification of the DE [19]. For that reason, the method of FTIR band deconvolution was used to calculate the DE. By infrared, it was observed that the pectin extracted in organic acid presented higher degree esterification, and it has a coherence with the zeta potential value, which was less negative with respect to the values obtained for the pectin extracted with inorganic acid. On the other hand, the TGA analysis showed a major decomposition temperature value for the pectin extracted with organic acid evidencing a possible higher content of esterified groups. It is observed that the drastic hydrolysis conditions proportioned by stronger acid allowed us to obtain pectin with lower degree esterification (<50%), possibly due to a degradation process generated during the hydrolysis, and then the uronic acid content is higher, so it has higher negative zeta potential values. These results are consistent with the extraction of pectin from passion fruit (*Passiflora edulis f. flavicarpa*) using nitric acid as extractant [35]. To study the effect of pectin properties on the polyelectrolyte formation, the pectin samples with low (2.4%, 24.3%) and high (96.8%) DE were selected for the subsequent studies.

### 3.3. Preparation and Characterization of Polyelectrolyte Nanoparticles

#### 3.3.1. Effect of Mass Ratio of AmCh:Pec

Table 2, Table 3 and Table 4 show in a specific way the data for the effect of mass ratio of the polyelectrolytes on particle size, zeta potential and polydispersity index (PdI). It is observed how the DE influences the electrostatic interaction between pectin and AmCh for the polyelectrolyte formation.

**Figure 4:**
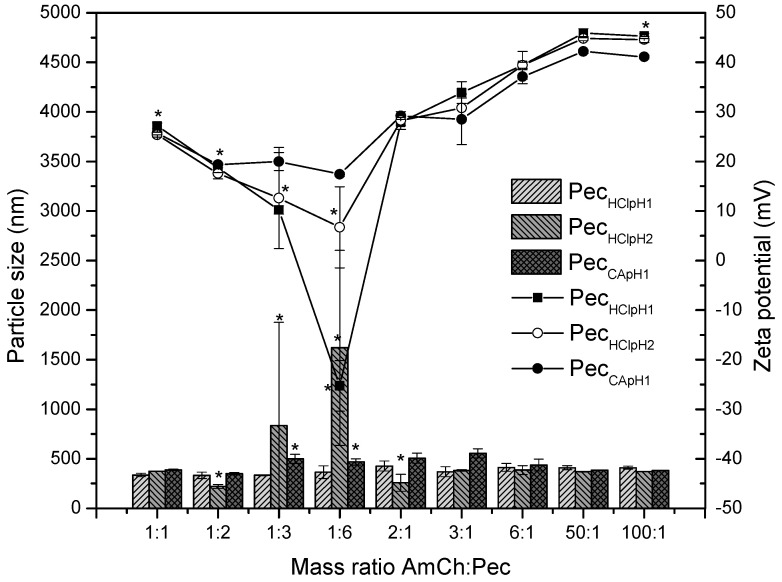
Effect of mass ratio of AmCh:Pec on the particle size and zeta potential using the pectin samples Pec_HClpH1_, Pec_HClpH2_ and Pec_CApH1_. Statistically significant: * *p* = 0.00.

The results of Table 2 and Figure 4 show the effect of mass ratio on the polyelectrolyte properties through the interaction between pectin with low degree of esterification (Pec_HClpH1_, pectin extracted with HCl at pH1) and AmCh. It was observed that the zeta potential decreases from 48.8 mV to −25.3 mV as the pectin mass increases due to pectin acting as a polyanion allowing an electrostatic interaction between its carboxylate groups (–COO^−^) and the protonated amino groups of the AmCh polymer (–NH_3_^+^). This interaction was evident since the zeta potential decreased with the increasing pectin concentration, and then increased from 27.9 mV to 45.3 when the concentration of AmCh polycation increased. As the mass amount of AmCh polymer increases, there are not enough carboxylate groups to interact with the -NH_3_^+^ groups, remaining an excess of positive charges on the nanoparticle surface [36]. On the other hand, the particle size decreased when the pectin concentration increased, because electrostatic interactions form complexes, and these interactions are strong to form particles. However, the polydispersity index increased when the pectin polyanion was added in excess (ratio 1:3 and 1:6) indicating that there was a limit of interaction between charges, making the interaction weak and the causing swelling of the particle. The interactions between polyelectrolytes must be strong to guarantee a surface charge on the polyelectrolyte complex which stabilizes the system by electrostatic repulsions and forming compact systems that does not allow water to flow inside (ratio 1:1, 1:2, 3:1, 6:1, 50:1, and 100:1) [37].

Table 3 and the Figure 4 show the effect on the polyelectrolyte properties causing different interactions between pectin and AmCh at different mass ratios when using pectin with other percent of low degree of esterification (Pec_HClpH2_, extracted with HCl at pH2). It was observed that the zeta potential decreased from 45.1 mV to 6.7 mV as the pectin mass increased. On the other hand, by increasing the amount of AmCh polycation concentration, the zeta potential increased from 25.4 mV to 44.6 mV. Regarding stability, it was observed that the PdI was favored in 1:1, 3:1, 6:1, 50:1, and 100:1 ratios. That is to say, pectin at high concentration does not have a good interaction with the AmCh due to the net charge of the polyelectrolyte being close to 0 mV, which does not allow electrostatic repulsions making the system unstable and forming heterogeneous particle size distributions and PdI increases [11,15].

The results of Table 4 and Figure 4 show the effect on the polyelectrolyte properties cause different interactions between pectin and AmCh at different mass ratios when using pectin with a high degree of esterification (Pec_CApH1_, pectin extracted with CA at pH1). It is observed that the zeta potential decreased from 44.8 mV to 17.4 mV as the pectin mass increased. On the other hand, at increasing concentrations of AmCh polycation, the zeta potential increased from 25.7 mV to 41.1 mV. The polyelectrolyte formation was favored in the ratios 1:1, 1:2, 3:1, 6:1, 50:1 and 100:1; due to the particle size distributions being more homogeneous at low concentrations of pectin and at higher concentrations of AmCh polycation, again due to electrostatic interactions being stronger under these conditions.

When comparing the three pectin samples forming polyelectrolyte complexes with AmCh, Figure 4, the most favorable systems were 1:1, 3:1, 6:1, 50:1 and 100:1, which showed homogeneous size distributions, adequate zeta potentials that contributed to the stability and a polydispersity index less than 0.3. However, pectin with 24.3% of esterification degree (Pec_HClpH2_, 24.3%) obtained slightly smaller particle sizes with average values of 370 nm meaning pectin with low DE, i.e., 24.3%, favors electrostatic interactions with cationic AmCh allowing a net charge to keep the stability by electrostatic repulsion and to form complexes. Pectin with low DE–2.4% did not favor the electrostatic interaction, due to excess of negative charges carrying a neutral surface charge, which avoids the stabilization of the polyelectrolyte by electrostatic repulsions [11,15,16,38,39,40]. In addition, the voluminous methoxy groups in pectin with high DE, i.e., 96.8%, also avoids electrostatic interaction, generating swelling processes and an increase in the particle size. It is concluded that, it is important to guarantee an adequate electrostatic interaction in order to get the formation of compact polyelectrolytes nanoparticles with particle size values of 370 nm and surface charge close to 25 mV or 40 mV. Pectin with degree of esterification 24.3% showed these properties. So, the next study was development using this pectin sample. 

#### 3.3.2. Effect of the AmCh and Pectin Concentration 

The results in Table 5 show that the smaller particle size, below 400 nm, was obtained at a lower concentration of AmCh (1 mg/mL) and a higher concentration of pectin (1 mg/mL). As the concentration of AmCh increased, the particle size increased since electrostatic interactions with the pectin are not strong enough to form dense complexes. So, water flow is generated inside the polyelectrolyte, causing swelling and the increasing particle size. The zeta potential results showed a net positive charge in all samples, and it could be hoped to see a negative charge at the same concentration of the polymers. However, this behavior possibly is due to the pectin polymer stabilizing the electrostatic interaction inside of the polyelectrolyte complexes, as it has been shown in other studies: the variation in particle size is evidence of electrostatic interaction. An increase in particle size was evident, for example, particle size from 390.8 to 452.5 nm, for 0.2 mg/mL concentration of pectin and AmCh 1.0 and 2.0 mg/mL, respectively, possibly due to the pectin polyanion diffusing into the nucleus of the nanoparticles and the interaction of charges occurring inside and so generating a positive charge in the polyelectrolytes [12]. In accordance with the above, all systems are stable and the PdI values were lower than 0.3; however, the system with a concentration of 1 mg/mL for both polymers was selected to evaluate the effect of pH and temperature on polyelectrolyte complex stability and particle size (372.8 nm), and for the next studies. 

#### 3.3.3. Effect of the Temperature and PH 

**Figure 5:**
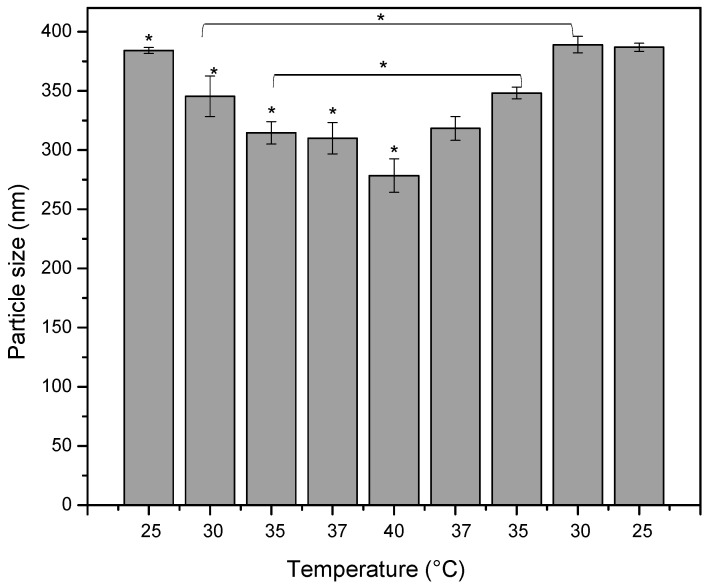
Effect of the temperature on the polyelectrolyte nanoparticles based on AmCh:Pec_HClpH2_ with concentration 1 mg/mL. Statistically significant: * *p* = 0.00.

Figure 5 shows that the particle size decreased from 384 ± 2 nm to 278 ± 14 nm, as the temperature increased from 25 to 40 °C, and the PdI values were close to 0.1. When the temperature was returned to the previous temperature values, particle size increased until reaching a value close to the initially obtained at that same temperature, from 278 ± 14 nm to 387 ± 4 nm, indicating a thermosensitive behavior. However, statistically some particle size values inside the temperature level did not present significative statistically differences, which did not lead to conclusions about thermosensitive behavior.

The decreasing particle size is due to the change of pectin behavior with temperature. In previous studies, it has been found that the viscosity of pectin decreased with increasing temperature, which would allow polymer chains to extend and the intermolecular distances to increase since there is an increase in the energy of the molecules, and this implies a thermal expansion [21]. When this happens, carboxylic acid groups are more exposed and allow greater electrostatic interactions with positively charged amino groups of AmCh polymer, forming a stronger complex and decreasing particle size [12]. The development of nanocarriers with properties such as temperature response is important due to the effect in the efficiency of the encapsulated molecule and for the preparation of smart drug delivery systems, for biomedical applications, as it was shown in the study of the delivering 5-FU encapsulated in a biodegradable thermo-responsive chitosan-g-poly(N-vinylcaprolactam) biopolymer composite for treatment of cancer cells, where the 5-FU drug release was found to be more prominent above of the phase transition temperature of the polymer (38 °C) [41]. 

**Figure 6:**
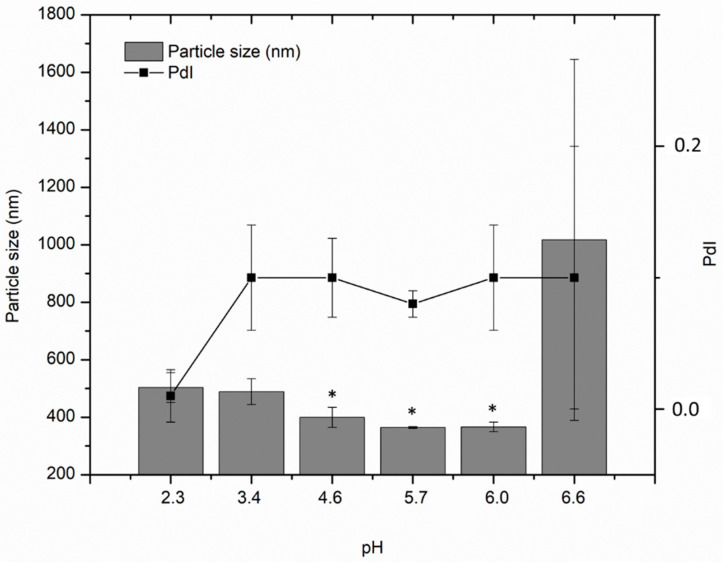
Effect of the pH on the polyelectrolyte nanoparticles based on AmCh:Pec_HClpH2_ with concentration 1 mg/mL. Statistically significant: * *p* = 0.03.

On the other hand, Figure 6 shows that particle size increased with the increasing pH. When the pH reached a value of 6.6, the chitosan–pectin polyelectrolytes were destabilized and formed microparticles with values close to 1000 nm. At pH 7.5 (not shown) there was not size distribution which indicated that there were free polymers in solution. In previous studies, it has been found that chitosan solutions at pH 4.0 reached a major positive value of zeta potential, in our study it was close to 38 mV and the pectins had a negative value close to −15 mV. This implies that both polymers had the availability of oppositely charged groups to form a complex by electrostatic interaction, so the interaction will be favored, and the final charge will stabilize the polyelectrolyte complex by electrostatic repulsions between them [11,16]. When the pH value increased, the chitosan presented a tendency to be less positive and the pectin charge became more negative, which led to the neutrality of the complex and its subsequent destabilization, as it has been evidenced in previous studies of the interaction of these particles of amphiphilic chitosan with hyaluronic acid of low molecular weight [20].

**Figure 7:**
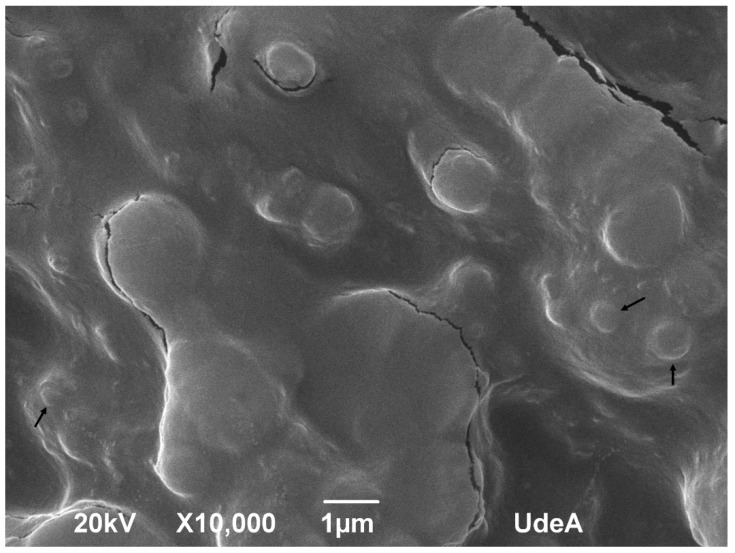
SEM image of polyelectrolyte nanoparticles based on AmCh:Pec_HClpH2_.

Figure 7 showed the SEM image of AmCh:Pec_HClpH2_ polyelectrolytes, the nanoparticles presented a semi-spherical form and the particle sizes showed values above 400 nm. This value can be due to the particles forming aggregates during sample preparation for analysis, which was subjected to a vacuum drying process. Previous studies have demonstrated that non-covalent interactions, such as hydrogen bonding and electrostatic interactions, favour the formation of aggregates or a packed matrix [18]. 

### 3.4. Encapsulation of Nile Red in Polyelectrolyte Nanoparticles

**Figure 8:**
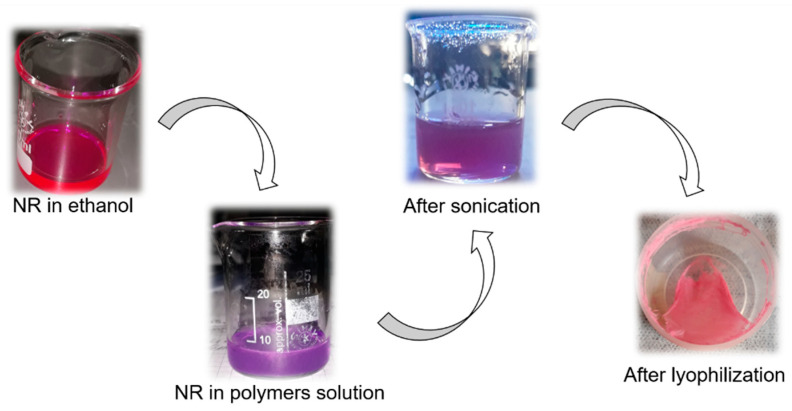
Behavior of NR in different media of interaction: NR in ethanol: fuchsia color. NR in aqueous solution of the polymers: purple color. After sonication: light purple color. After lyophilization process: light pink color.

As polyelectrolyte complexes were made from polymers with amphiphilic nature, in the present work a proof molecule such as Nile Red (NR) was used, which is a hydrophobic molecule and solvatochromic fluorophore with different behavior in polar and non-polar environments. The fluorescent properties are strongly influenced by the medium in which it is dissolved. In polar solvents, such as methanol, it has a purple color, under visible light. In ethanol, it takes a fuchsia color. In aprotic polar solvents, such as acetone, acetonitrile, and ethyl acetate, it takes a light pink color. So, the color of the NR changes from a tone of pink color until yellow tone when the polarity of the medium decreases [42,43]. As shown in Figure 8, NR was dissolved in ethanol and when it was added to the aqueous acid solution of the polymers, the mixture turned a purple color due to the polarity increased. After sonication, it had a lighter purple color but during the ultrafiltration the color turned slightly pink which could indicate that the NR interacted with a non-polar environment. After the lyophilization process, the solid had a pink color, and this implies that NR interacted with the non-polar environment of the polyelectrolyte complex. The results agree with previous studies, such as the encapsulation of the NR in PLA/Pluronic nanoparticles [26], in nanostructures of non-ionic amphiphiles using 2,2-di(prop-2-yn-1-yl)propane-1,3-dio [44] and a sensor based on fluorescent dye-doped [45].

The NR was added in different mass ratios with respect to the AmCh:Pectin polymer. Table 6 shows that by increasing the amount of NR, the encapsulation efficiency and loading capacity increased, achieving a maximum load capacity of 1.8% and an encapsulation efficiency of 80%. In addition, the recovery percentage indicated that the ultrafiltration method is suitable for purifying the polyelectrolytes, since on average 90% of the prepared polyelectrolytes were recuperated. Since chitosan is an amphiphilic polymer, with this property provided by modification with oleic acid, it allows for the encapsulation of hydrophobic molecules.

## 4. Conclusions

Pectin from banana peels *Musa paradisiaca* is extracted with higher yield using citric acid. Hydrochloride acid at pH 1 and 2 allows isolation of pectin with a low degree of esterification. The polyelectrolyte complexes obtained are through electrostatic interactions between polyanion pectin with a low degree of esterification (24.3%) and the AmCh polycation, which is shown by changes in the zeta potential and homogenous particle size distributions at 370 nm. The stable polyelectrolyte complexes present a net surface charge close to 25–40 mV, and for that, the mass ratio of the polyelectrolytes AmCh:Pec is at least equal, or the polycation AmCh can be added in higher proportion. The adequate polymer concentrations are 1 mg/mL. Polyelectrolyte nanoparticles based on AmCh:Pec show a semi-spherical form with smallest particles size observed at 40 °C. These systems are stable at pH 5–6 and demonstrate a capacity to encapsulate hydrophobic molecules, offering an alternative as a drug carrier system. Further investigations are necessary to determine the drug delivery behavior under physiological conditions. 

## Figures and Tables

**Table 1:** Effect of acid type and pH on pectin properties.

Sample	Extraction Yield ± SD (%)	DE ± SD (%)	Zeta Potential ± SD (mV)
**Pec_HClpH1_**	7.8 ± 0.8 *	2.4 ± 0.2 *	−28.6 ± 1.6 *
**Pec_HClpH2_**	6.2 ± 0.2 *	24.3 ± 0.7 *	−34.8 ± 0.9 *
**Pec_CApH1_**	23.9 ± 1.0 *	96.8 ± 3.1 *	−11.8 ± 0.3 *
**Pec_CApH2_**	18.1 ± 0.9 *	34.2 ± 1.0 *	−9.8 ± 1.3 *

Data represent the mean ± SD (n = 3). Statistically significant: * *p* = 0.00.

**Table 2:** Effect of mass ratio of AmCh:Pec_HClpH1_ on the particle size and PdI of the polyelectrolyte nanoparticles.

Polymer	AmCh: Pec_HClpH1_	Particle size (nm) ± SD	PdI ± SD
**Constant AmCh mass**	1:1	336.6 ± 17.0 *	0.2 ± 0.1
	1:2	333.7 ± 33.3 *	0.2 ± 0.1
	1:3	335.2 ± 3.6 *	0.8 ± 0.2 **
	1:6	364.4 ± 64.8	1.4 ± 1.8 **
**Constant Pec_HClpH1_ mass**	2:1	426.7 ± 49.6	0.3 ± 0.3
	3:1	369.5 ± 51.0	0.2 ± 0.4
	6:1	412.5 ± 39.4	0.1 ± 0.0
	50:1	410.3 ± 20.4	0.1 ± 0.0
	100:1	410.7 ± 15.3	0.1 ± 0.0

AmCh: amphiphilic chitosan; Pec_HClpH1_: Pectin extracted with HCl at pH1; PdI: polydispersity index; SD: standard deviation. Data represents the mean ± SD (n = 3). Statistically significant: * *p* = 0.02, ** *p* = 0.03.

**Table 3:** Effect of mass ratio of AmCh:Pec_HClpH2_ on the particle size and PdI of the polyelectrolyte nanoparticles.

Polymer	AmCh: Pec_HClpH2_	Particle Size (nm) ± SD	PdI ± SD
**Constant AmCh mass**	1:1	372.8 ± 2.2	0.1 ± 0.0 **
	1:2	221 ± 19.9	0.6 ± 0.4 **
	1:3	836.1 ± 1041.5	0.8 ± 1.0 **
	1:6	1620.1 ± 983.9 *	0.1 ± 0.1
**Constant Pec_HClpH2_ mass**	2:1	258.2 ± 86.5	1.3 ± 0.2 **
	3:1	382.0 ± 7.7	0.3 ± 0.0
	6:1	386.8 ± 45.7	0.2 ± 0.0
	50:1	370.2 ± 4.3	0.1 ± 0.0
	100:1	370.5 ± 1.2	0.1 ± 0.0

AmCh: amphiphilic chitosan; Pec_HClpH2_: Pectin extracted with HCl at pH2; PdI: polydispersity; SD: standard deviation. Data represents the mean ± SD (n = 3). Statistically significant: * *p* = 0.03, ** *p* = 0.00.

**Table 4:** Effect of mass ratio of AmCh:Pec_CApH1_ on the particle size and PdI of the polyelectrolyte nanoparticles.

Polymer	AmCh: Pec_CApH1_	Particle Size (nm) ± SD	PdI ± SD
**Constant AmCh mass**	1:1	387.7 ± 6.7 *	0.2 ± 0.1
	1:2	350.3 ± 12.7 *	0.2 ± 0.1
	1:3	499.4 ± 47.5 *	0.2 ± 0.2
	1:6	469.2 ± 31.2 *	0.2 ± 0.1
**Constant Pec_CApH1_ mass**	2:1	506.1 ± 50.7 *	0.3 ± 0.4
	3:1	555.2 ± 46.1 *	0.1 ± 0.0
	6:1	435.6 ± 60.3	0.1 ± 0.1
	50:1	384.7 ± 3.2	0.1 ± 0.0
	100:1	382.5 ± 2.4	0.1 ± 0.0

AmCh: amphiphilic chitosan; Pec_CApH1_: Pectin extracted with CA at pH1; PdI: polydispersity; SD: standard deviation. Data represents the mean ± SD (n = 3). Statistically significant: * *p* = 0.01.

**Table 5:** Effect of the polymer concentration (AmCh and Pec_HClpH2_) on the particle size, PdI and zeta potential of the polyelectrolyte nanoparticles.

AmCh Concentration (mg/mL)	Pec_HClpH2_ Concentration (mg/mL)	Particle Size (nm) ± SD	PdI ± SD	Zeta Potential (mV) ± SD
1.0	0.2	390.8 ± 10.3 *	0.1 ± 0.03	36.3 ± 0.8 *
	0.5	385.0 ± 1.4	0.2 ± 0.03 **	31.6 ± 0.3 *
	1.0	372.8 ± 2.2	0.1 ± 0.0	25.4 ± 0.5 *
1.5	0.2	420.0 ± 5.6	0.1 ± 0.01	37.2 ± 0.1
	0.5	432.5 ± 12.4 *	0.1 ± 0.01	34.4 ± 0.6 *
	1.0	435.6 ± 60.3	0.1 ± 0.1	29.1 ± 1.4 *
2.0	0.2	452.5 ± 9.8	0.1 ± 0.02	38.4 ± 0.2 *
	0.5	454.1 ± 14.9 *	0.2 ± 0.01	34.0 ± 0.4
	1.0	436.7 ± 49.6	0.3 ± 0.02 **	30.9 ± 0.6

AmCh: amphiphilic chitosan; Pec_HClpH2_: Pectin extracted with HCl to pH2; PdI: polydispersity; SD: standard deviation. Data represents the mean ± SD (n = 3). Statistically significant: * *p* = 0.00, ** *p* = 0.02.

**Table 6:** Relation mass NR/polymers, mean particle size, and encapsulation parameters of NR-loaded in polyelectrolyte nanoparticles of AmCh:Pec_HClpH2_.

Sample	NR Mass (µg)	Particle Size (nm) ± *SD*	PdI ± *SD*	EE ± *SD (%)*	LC ± *SD (%)*
**AmCh:Pec_HClpH2_**	0	345 ± 9	0.06 ± 0.02	-	-
**AmCh:Pec_HClpH2_/NR_2%_**	150	348 ± 10	0.08 ± 0.01 *	80 ± 8 **	1.8 ± 0.5 **
**AmCh:Pec_HClpH2_/NR_1%_**	70	341 ± 4	0.05 ± 0.01 *	65 ± 3 **	1.4 ± 0.3
**AmCh:Pec_HClpH2_/NR_0.5%_**	35	354 ± 3	0.15 ± 0.01 *	55 ± 3 **	1.2 ± 0.1

Data represents the mean ± SD (n = 3). EE: encapsulation efficiency. LC: loading capacity. Statistically significant: * *p* = 0.00, ** *p* = 0.02.
